# Mechanics and Composition of Middle Cerebral Arteries from Simulated Microgravity Rats with and without 1-h/d –Gx Gravitation

**DOI:** 10.1371/journal.pone.0097737

**Published:** 2014-05-19

**Authors:** Jiu-Hua Cheng, Li-Fan Zhang, Fang Gao, Yun-Gang Bai, Marco Boscolo, Xiao-Feng Huang, Xiang Zhang

**Affiliations:** 1 Department of Aerospace Physiology and Key Laboratory of Aerospace Medicine of Ministry of Education, Fourth Military Medical University, Xi'an, China; 2 Central Laboratory, School of Basic Medical Sciences, Fourth Military Medical University, Xi'an, China; 3 Department of Aerospace Engineering, Cranfield University, Cranfield, United Kingdom; Medical University of Graz, Austria

## Abstract

**Background:**

To elucidate further from the biomechanical aspect whether microgravity-induced cerebral vascular mal-adaptation might be a contributing factor to postflight orthostatic intolerance and the underlying mechanism accounting for the potential effectiveness of intermittent artificial gravity (IAG) in preventing this adverse effect.

**Methodology/Principal Findings:**

Middle cerebral arteries (MCAs) were isolated from 28-day SUS (tail-suspended, head-down tilt rats to simulate microgravity effect), S+D (SUS plus 1-h/d −G_x_ gravitation by normal standing to simulate IAG), and CON (control) rats. Vascular myogenic reactivity and circumferential stress-strain and axial force-pressure relationships and overall stiffness were examined using pressure arteriography and calculated. Acellular matrix components were quantified by electron microscopy. The results demonstrate that myogenic reactivity is susceptible to previous pressure-induced, serial constrictions. During the first-run of pressure increments, active MCAs from SUS rats can strongly stiffen their wall and maintain the vessels at very low strains, which can be prevented by the simulated IAG countermeasure. The strains are 0.03 and 0.14 respectively for SUS and S+D, while circumferential stress being kept at 0.5 (10^6^ dyn/cm^2^). During the second-run pressure steps, both the myogenic reactivity and active stiffness of the three groups declined. The distensibility of passive MCAs from S+D is significantly higher than CON and SUS, which may help to attenuate the vasodilatation impairment at low levels of pressure. Collagen and elastin percentages were increased and decreased, respectively, in MCAs from SUS and S+D as compared with CON; however, elastin was higher in S+D than SUS rats.

**Conclusions:**

Susceptibility to previous myogenic constrictions seems to be a self-limiting protective mechanism in cerebral small resistance arteries to prevent undue cerebral vasoconstriction during orthostasis at 1-G environment. Alleviating of active stiffening and increasing of distensibility of cerebral resistance arteries may underlie the countermeasure effectiveness of IAG.

## Introduction

Postflight orthostatic intolerance (POI) is a significant cardiovascular risk associated with spaceflight reentry and effective countermeasures are still incomplete [1]–[5]. Recent studies indicate that, in addition to hypovolemia and cardiac deconditioning, adaptation of arterial vessels to microgravity during spaceflight might be among the most important mechanisms responsible for POI [3]–[4], [6]–[8]. Inability to adequately elevate the total peripheral resistance has been identified as an important factor in the genesis of POI, whereas splanchnic and lower limb muscular vascular beds are the main contributors to the maintenance of peripheral resistance [3]–[4], [6]–[7]. Besides, enhanced cerebral vasoconstriction and/or impaired autoregulation that develops due to adaptation to microgravity during long-duration spaceflight might be another contributing factor [2], [7], [9]–[11]. Few of returning astronauts had significant symptoms and could not remain standing, but did not exhibit hypotension [7]. Furthermore, currently used, exercise-based countermeasures seem insufficient to prevent this cardiovascular dysfunction in future long-duration, planetary missions. In the past two decades, intermittent artificial gravity (IAG) by incorporating a short-arm centrifuge into the spacecraft has been suggested as a gravity-based countermeasure for future spaceflight and ground-based studies using intermittent centrifugation and exercise within lower body negative pressure (LBNP) have provided promising data [1], [4]–[5], [12]–[14].

In ground animal studies, the adaptation of cerebral arteries of tail-suspended, head-down tilt rats (SUS), which simulate cardiovascular effects of microgravity, are consistent with those reported for hypertensive rats [15]–[17]. And it has also been demonstrated that these adaptation changes are triggered and maintained by a sustained increase of transmural pressure (TMP), but not increase of flow and volume, in the cerebral vessels [4], [18]–[19]. In addition, our work has shown further that 1 h/d –G_x_ (dorsoventral) gravitation by restoring the rat's orthostatic standing posture and hence the normal TMP distribution along the arterial vasculature (S+D), which mimics the IAG countermeasure, is surprisingly effective in preventing hypertropic remodeling in cerebral arteries [20]–[22]. Nevertheless, the adaptation changes yet exhibits regional differences along different arterial segments in cerebral circulation. As we have shown, in the case of basilar artery, a large cerebral artery that also contributes importantly to cerebrovascular resistance [23], S+D can prevent both the enhanced vasoreactivity and hypertrophic remodeling that might occur due to SUS alone [22]; however, in MCA, a proximal resistance artery, it can prevent only hypertrophy but not the enhanced myogenic tone and increased vasoreactivity [21], [24]. To evaluate further the countermeasure effectiveness of IAG on cerebral small resistance arteries, it is also essential to understand their active and passive mechanical properties. Active biomechanical behavior of contracted arteries provides an understanding of their distensibility during smooth muscle cell activation and thereby how it would take part in controlling local blood flow in vivo. Passive mechanical properties provide an understanding of the changes of elasticity in MCAs, associated with remodeling changes (e.g., collagen and elastin) [25]–[26]. Specifically, in cerebral circulation, an increased distensibility of resistance arteries at low levels of pressure may help to preserve vasodilation capacity during hypotension [27]. In addition, it is also necessary to examine whether the myogenic mechanism in the MCA is susceptible to previous pressure-induced constrictions, because the biomechanical analysis is based on the pressure-diameter data thus obtained and we also need to know whether the cerebral resistance arteries do have such a self-limiting mechanism to protect them from severe and sustained constrictions.

The purpose of the present study was threefold: (1) to examine exclusively the myogenic reactivity to 2 runs of pressure increments for MCAs isolated from rats subjected to simulated microgravity for 28 days with and without daily 1-h –Gx intervention; (2) to evaluate and characterize the active mechanical behavior of the MCAs from different groups; and (3) to calculate the passive mechanical properties of these vessels and to measure the percentage of collagen and elastin in the vessel wall.

## Materials and Methods

### Ethical Approval

The procedures used in this study were approved by the Animal Care and Use Committee of Fourth Military Medical University and conform to the *Guide for the Care and Use of Laboratory Animals* published by the US National Institutes of Health (NIH Publication No. 85–23, revised 1996).

### Animal Model

#### Tail-suspended, head-down tilt rat model

The technique of tail suspension with modification from our laboratory has been described in detail previously [22], [28]. Briefly, the rats were maintained in an about −30° head-down tilt position with their hindlimbs unloaded to simulate the cardiovascular effect of microgravity. The controls were housed in identical Plexiglas cages, except that the tail suspension device was removed.

#### Model of daily short-duration –G_x_ gravitation

Daily stationary ground support in normal standing (STD) posture for 1 h was adopted to simulate the countermeasure effect of IAG as previously described [22], [29]. Briefly, the suspended rat was released from suspension and then placed into a 50-cm-long, tube-like metallic mesh cage maintained in horizontal position for 1 h. The rat could move forward and backward, but it could not turn around. The gravity vector was –G_x_.

### Experimental Design

In *protocol 1*, pressure-diameter relationship of MCAs in active and passive states to a stepwise pressure increase from 4–175 mmHg was determined [30]; it was measured twice for each vessel segment in an arteriograph. Since it is difficult to measure at the 0 mmHg level accurately, initial pressure of 4 mmHg was used to represent the nominal zero value. Incremental steps were kept at 25 mmHg thereafter, except for the first increment which was 21 mmHg. Subsequently, the active and passive circumferential stress (σ_θ_) - strain (ε_θ_) relationships and the passive stiffness parameter (β) were calculated. Male Sprague-Dawley rats were randomly assigned to three experimental groups (*n* = 13 rats/group): control (CON), tail suspension (SUS), and daily suspension for 23 h plus standing for 1 h (S+D). During the 28-day period, daily STD intervention was conducted between 08:00 and 09:00.

In *protocol 2*, wall composition of the MCAs from SUS, S+D, and CON rats was measured by electron microscopy and compared. The ultrathin longitudinal middlemost sections specially prepared in our previous study [21] for morphometry of wall histology were reused under a higher magnification to measure the percentage of area occupied by collagen and elastin in the vessel wall area scanned.

### Experimental Procedure

#### Pressure arteriography

After 28 days of simulation, rats were anesthetized and the MCAs were isolated and tied onto two glass micropipettes of the vessel chamber of the arteriograph as previously described [21]. After cannulation, each isolated vessel in the chamber was transferred to the stage of an inverted microscope coupled to a video camera, a video micrometer, and a data acquisition system (Pressure Myograph System P110, DMT, Denmark). The procedure for equilibration, setting the arterial axial length to its in-vivo length, removing buckling, and reducing mechanical hysteresis has been described in our previous study [21].

To fully evaluate the in vitro myogenic behavior of MCAs, intraluminal pressure was increased from 4 to 175 mmHg [30]. Each step was maintained for 5−10 min to allow the vessel to reach a steady-state diameter and vessel diameter and longitudinal force were registered. To examine the repeatability of responses in different phases [30], after the first run of pressure steps, the superfusion physiological saline solution (PSS) was replaced and equilibrated for 15 min, and then the second run was repeated. Finally, a passive pressure-diameter relationship was obtained by incubating the arterial segment with Ca^2+^-free PSS containing 2 mM ethyleneglycol-bis(*β*-aminoethylether)-*N*, *N'*-tetraacetic acid (EGTA) for 30 min and repeating the pressure schedule twice for each vessel. Only the second-run data were used to calculate the passive mechanical properties, since passive arteries exhibit a nearly repeatable response to cyclic loading once they have been preconditioned [26].

The myogenic tone was calculated as:



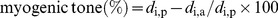
(1)where *d*_i,p_ is the passive internal diameter determined in Ca^2+^-free PSS and *d*_i,a_ is the active internal diameter at a particular intraluminal pressure.

#### Collagen and elastin measurement

Electron micrographs were taken at an original magnification of ×4,000 with a JEM-2000EX transmission electron microscope (JEOL, Tokyo, Japan) and enlarged by a factor of 3.75 for a final magnification of ×15,000. The areas of media (M) and adventitia (A) were measured and calculated, and then the areas occupied by collagen and elastin fibrils, respectively, in M and A were measured by repeated tracing the edges of the profiles of these acellular components sectioned along different orientations in the electron micrographs by an image analysis software (Image-Pro Plus 6.0) as described by Intengan et al. [31]. Then the percentage of area occupied by collagen and elastin in M and A was calculated respectively. To insure uniform sampling, the quantification of the percentage of collagen and elastin was made randomly for a total of 4 or 5 non-overlapping micrographs from each vessel by two observers.

### Modeling and Calculations

In this study, the rat MCA is considered to be a thick-walled cylindrical tube with orthotropic elasticity and the wall material is assumed to be incompressible, homogeneous, and globally nonlinear [25].

#### Circumferential stress (σ_θ_) - strain (ε_θ_) relationship

Because the thin-wall assumption, i.e., the ratio of wall thickness to internal radius <0.1, is not valid for these vessels, Laplace's equation was not adopted for calculating the vessel-wall stress. The circumferential stress (σ_θ_) was calculated at the internal and external radius using the equation originally derived by Timoshenko for a hollow cylinder submitted to uniform pressure [32]

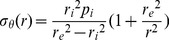
(2)where 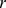
is the radius, *r*_i_ the internal radius, *r*_e_ the external radius, and p_i_ the internal pressure.

According to the definition of strain, the circumferential strain (ε_θ_) at the internal and external radius was calculated by:
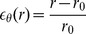
(3)where *r*_0_ is the radius at zero pressure (actually p_i_ was set at 4 mmHg).

#### Overall stiffness parameter (β)

β was calculated for the passive vessels by a curve-fitting technique. The logarithmic transformation of the p-d data and linear regression to find β was performed using the following equation [33].
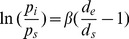
(4)where p_i_ is the internal pressure, p_s_ is a reference pressure chosen in the physiological pressure range, *d*_e_ the external diameter, and *d*_s_ is the external diameter at the reference pressure. A reference pressure of 75 mmHg was chosen because it lies within the physiological pressure range and gave a good fit to the data (mean *R* ^2^>0.90).

### Statistical Analysis

Values are means ± SE. One vessel per rat was used for analyses. Model fits were considered highly correlated for *R* ^2^>0.90. A two-way ANOVA with repeated measures was used to detect the differences of the p-d relationship within (pressures) and between (groups) factors and the Student-Newman-Keuls *post hoc* test was used to determine the significance of differences among means; and then a three-way ANOVA was supplemented to evaluate further the difference between the two Runs. A one-way ANOVA was used to compare β values among the three groups, with the LSD *post hoc* test for multiple comparisons. Student's *t*-tests were used to determine whether differences in body mass, soleus muscle (wet) mass, myogenic tone (%) and dimensions of vessel segments at a particular pressure of 100 mmHg, percentage of acellular matrix components, and axial force were significant between groups. A value of *P*<0.05 was considered statistically significant. Statistical analysis was performed with the SPSS 9.0 package.

## Results

The data of body mass and soleus muscle (wet) mass are summarized in Table 1. There were no significant differences in final body mass among the three groups of *protocol 1*. The soleus muscle mass of SUS rats was 61% less than that of the CON rats (*P*<0.01). However, the soleus muscle mass of S+D rats was 37% less than CON rats (*P*<0.01), indicating the countermeasure effectiveness of 1 h/day STD in attenuating muscle atrophy [29].

**Table 1:** Body mass and left soleus muscle (wet) mass of CON, SUS, and S+D rats in Protocol 1.

	Body mass, g		Left soleus muscle mass	
	Initial	Final	Absolute, mg	Relative, mg/g body mass
CON	305.2±7.4	449.5±7.6	173.1±4.7	0.39±0.01
SUS	312.8±8.8	424.2±12.3	67.7±4.4**	0.16±0.01**
S+D	307.3±8.0	425.5±5.7	109.2±3.2** ##	0.26±0.01** ##

Values are means±SE, *n* = 13 animals/group. CON, control; SUS, 4-wk tail-suspended; S+D, 23 h/day suspension +1 h/day standing.

**P<0.01 vs. CON;

## *P*<0.01 vs. SUS.

### Vascular mechanics

#### Vessel characteristics

The external and internal diameters in active and passive states at 100 mmHg in the two runs of pressure steps are summarized in Table 2. In the first run, the passive diameters (d_e,p_ and d_i,p_) of CON at 100 mmHg were significantly greater (*P*<0.01) than SUS vessels. However, the passive wall thickness (Wp) of MCAs from SUS was significantly greater (*P*<0.01) than CON vessels. The active diameters (d_e,a_ and d_i,a_) of SUS and S+D at 100 mmHg were significantly smaller (*P*<0.01) than CON vessels. Hence the myogenic tone was significantly greater (*P*<0.01) in SUS and S+D than CON vessels.

**Table 2:** Dimensions of segments of middle cerebral arteries isolated from CON, SUS, and S+D rats (unit: µm).

	Vessel data at 100 mmHg			Group comparison		
	CON	SUS	S+D	CON vs. SUS	SUS vs.S+D	CON vs. S+D
			*First run (n = 13)*			
** d_e, p_**	270.4±6.2	247.9±4.0	256.3±7.4	**	NS	NS
** d_e, a_**	199.5±10.0	161.3±3.1	165.5±7.2	**	NS	**
** d_i, p_**	245.3±5.7	219.2±4.3	230.3±7.2	**	NS	NS
** d_i, a_**	169.3±10.4	128.9±4.2	134.3±7.8	**	NS	**
** W_p_**	12.5±0.5	14.4±0.5	13.0±0.6	**	NS	NS
** W_a_**	15.1±0.9	16.2±1.0	15.6±0.6	NS	NS	NS
Tone (**%**)	31.3±3.4	41.0±1.8	41.8±2.5	**	NS	**
			*Second run (n = 8∼10)*			
** d_e, p_**	267.7±5.8	250.0±4.3	262.1±7.9	*	NS	NS
** d_e, a_**	213.8±8.9	187.1±4.6§§	200.6±14.4§	*	NS	NS
** d_i, p_**	244.2±5.7	223.2±4.8	237.2±7.7	*	NS	NS
** d_i, a_**	186.4±8.5	158.4±5.7§§	173.3±15.3	NS	NS	NS
** W_p_**	11.8±0.5	13.4±0.4	12.5±0.5	*	NS	NS
** W_a_**	13.7±0.6	14.4±0.8	13.7±0.9	NS	NS	NS
Tone (**%**)	23.7±2.8	28.6±3.1	27.1±5.5	NS	NS	NS

Measurements were made on cannulated vessels pressurized to 100 mmHg. *n* =  number of animals/group. d_e_, external diameter; d_i_, internal diameter; W, wall thickness  = 1/2(d_e_–d_i_); Subscripts *p* and *a* represent passive and active state, respectively. NS, not significant;

*P<0.05,

**P<0.01.

#### Pressure-diameter relationship

**Figure 1:**
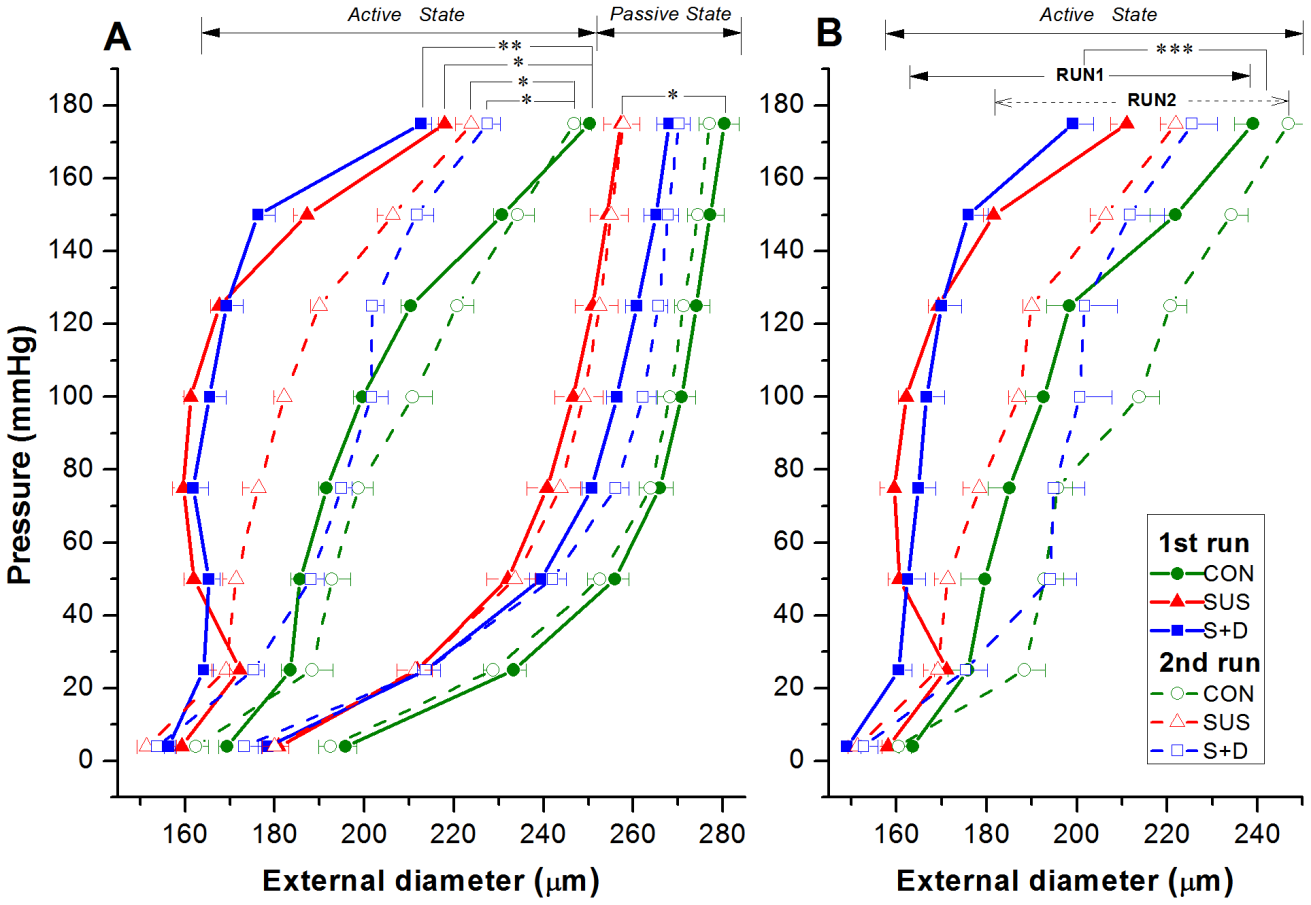
(A) Pressure-external diameter relationship of middle cerebral arteries isolated from control (CON), 4-wk suspended (SUS), and 23-h/day suspended + 1-h/day standing (S+D) rats in active and passive states within the intraluminal pressure range of 4∼175 mmHg. Two separate runs of pressure steps were performed. Data from the first and second run are represented by filled symbols connected by thick lines and open symbols by dashed lines, respectively. Values are means ± SE. Active state: 1st run, *n* = 13 per group; 2nd run, *n* = 8∼10 per group. Passive state: *n* = 13 per group for both runs. *P<0.05, **P<0.01 (2-way ANOVA). (B) Evaluation of the difference in active pressure-external diameter relationship between the 1st and 2nd run using a three-way ANOVA. *n* = 8∼10 per group. *P*<0.001, F = 32.9, df = 1, 24.

Figure 1A shows the active and passive pressure (p)-external diameter (d_e_) relationships of MCAs from the three groups during the first and second runs of pressure steps. In passive state, the p-d_e_ relationship displayed an exponential curve in the pressure range of 4–175 mmHg: diameter increased sharply at lower pressures and then at much reduced increment rate at higher pressures. Although the p-d_e_ curves for the two runs were quite similar for each group, the passive p-d_e_ relationships were significantly different among the three groups and the significance of these differences being further confirmed by statistical analysis on the first run data (*P*<0.05). In active state, external diameters of MCAs from the three groups were much smaller than passive diameters due to the presence of myogenic tone and reactivity. The active p-d_e_ data did not fit an exponential relationship and displayed an irregularly shaped curve. During the first run of pressure steps, between 50 and 125 mmHg, MCAs from SUS and S+D maintained an essentially constant and significantly smaller diameter compared with that of CON rats (*P*<0.01); however, between 125 and 175 mmHg, the diameter increased steadily with increasing pressure but still remained significantly smaller than that of CON rats (*P*<0.05). Whereas the p-d_e_ curve of the CON group was situated rightward and displayed a monotonic increase of diameter with increasing pressure. However, during the second run of pressure steps, all the active p-d_e_ curves shifted rightward with the extent of shift being greatest in S+D, less in SUS, and least in CON group, which indicates that the decline in myogenic reactivity after the first series was most prominent in MCAs from S+D rats. Nevertheless, at 175 mmHg, the diameter of SUS and S+D remained significantly smaller than CON group (*P*<0.01). Figure 1B shows that the difference between the 1st and 2nd run was highly significant (three-way ANOVA; *P*<0.001).

#### Active mechanical behavior

**Figure 2:**
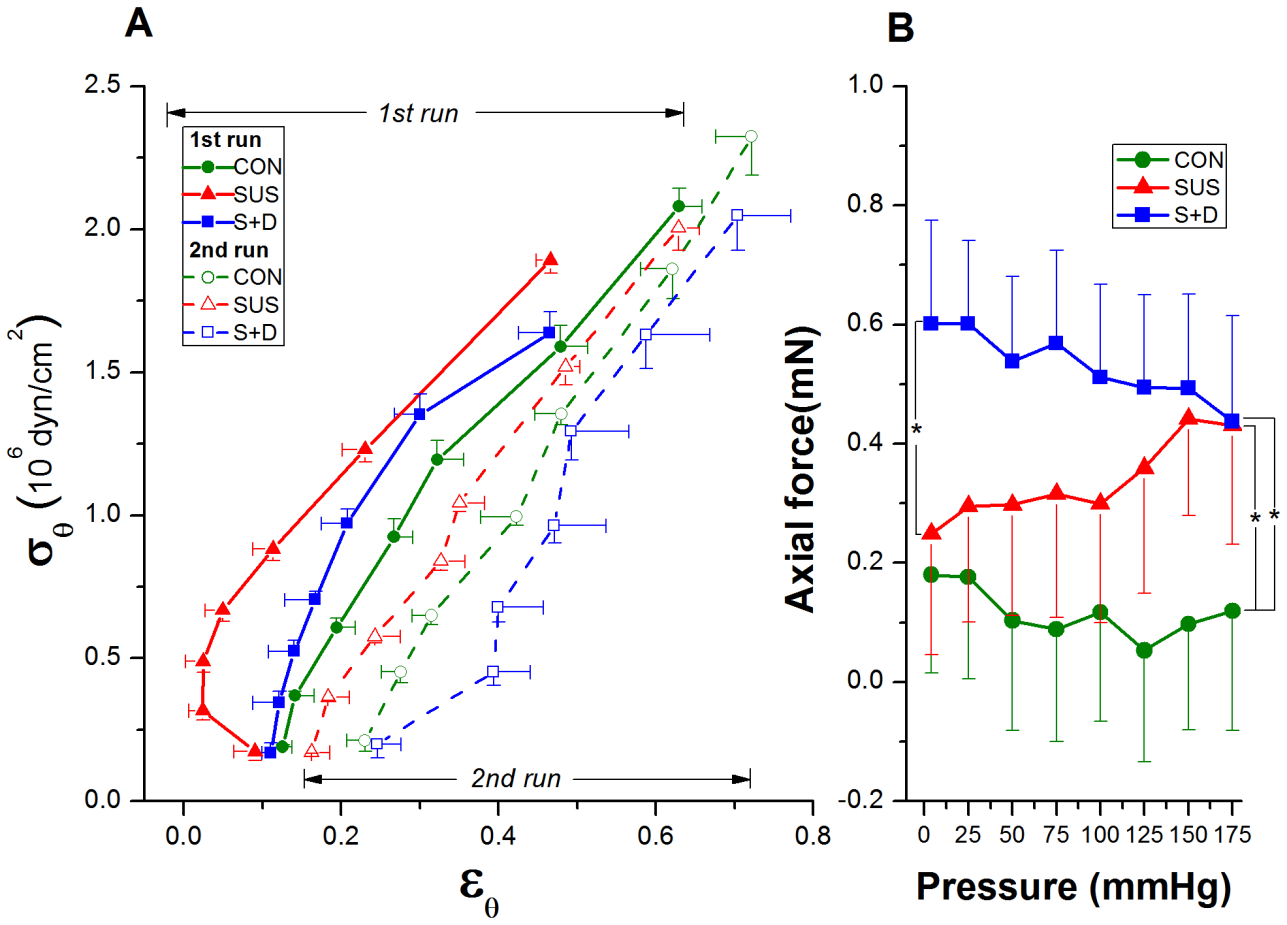
(A) Active circumferential stress (σ_θ_)-strain (ε_θ_) relationship of middle cerebral arteries isolated from CON, SUS, and S+D rats. Both σ_θ_ and ε_θ_ are calculated at the internal radius. The relationships calculated from the data of the first and second runs are depicted by curves drawn in thick lines with filled symbols and in dashed lines with open symbols, respectively. Values are means ± SE. *n* = 8∼10 per group. (B) Axial force-pressure relationship of middle cerebral arteries isolated from CON, SUS, and S+D rats in active state within the intraluminal pressure range of 4∼175 mmHg. The calculation was based on the data of the first run. Values are means ± SE. *n* = 13 per group. *P<0.05 (*t*-test).

Figure 2A shows the circumferential stress (σ_θ_)-strain (ε_θ_) relationship, which did not fit an exponential relationship and behaved much differently among the three groups and between the two runs of each group. In the first run, the active σ_θ_–ε_θ_ relationship of MCAs from SUS shifted leftward compared to that of S+D and CON vessels and the strain greatly decreased between 50 and 125 mmHg generated an almost parabolic σ_θ_–ε_θ_ curve within the myogenic pressure range. And the σ_θ_–ε_θ_ curves of S+D and CON groups were like two nearly parallel lines. However, at the highest stress corresponding to the pressure step of 175 mmHg, the vessel strain of SUS and S+D group remained smaller than that of the CON rats (Figure 2A, thick lines). In the second run, the σ_θ_–ε_θ_ relationships of MCAs from all the three groups shifted rightward indicating an overall decline in the performance of the myogenic mechanism after the first run of pressure-induced constrictions. The active σ_θ_–ε_θ_ curve of SUS group did not display the shape of a parabola any more. And the active σ_θ_–ε_θ_ curve of S+D shifted rightward to the CON curve, indicating a greater strain in the myogenic range than CON (Figure 2A, dashed lines).

Fig. 2B presents axial force-pressure data for the three groups. It shows that the force acting on the CON vessel is almost constant, whereas the axial forces on the SUS and S+D vessels tends to increase or decrease with the increasing pressure. Although the within-group variation is not significant, differences at 4 and 175 mmHg among the three groups are significant (*t*-test, *P*<0.05).

#### Passive mechanical property

**Figure 3:**
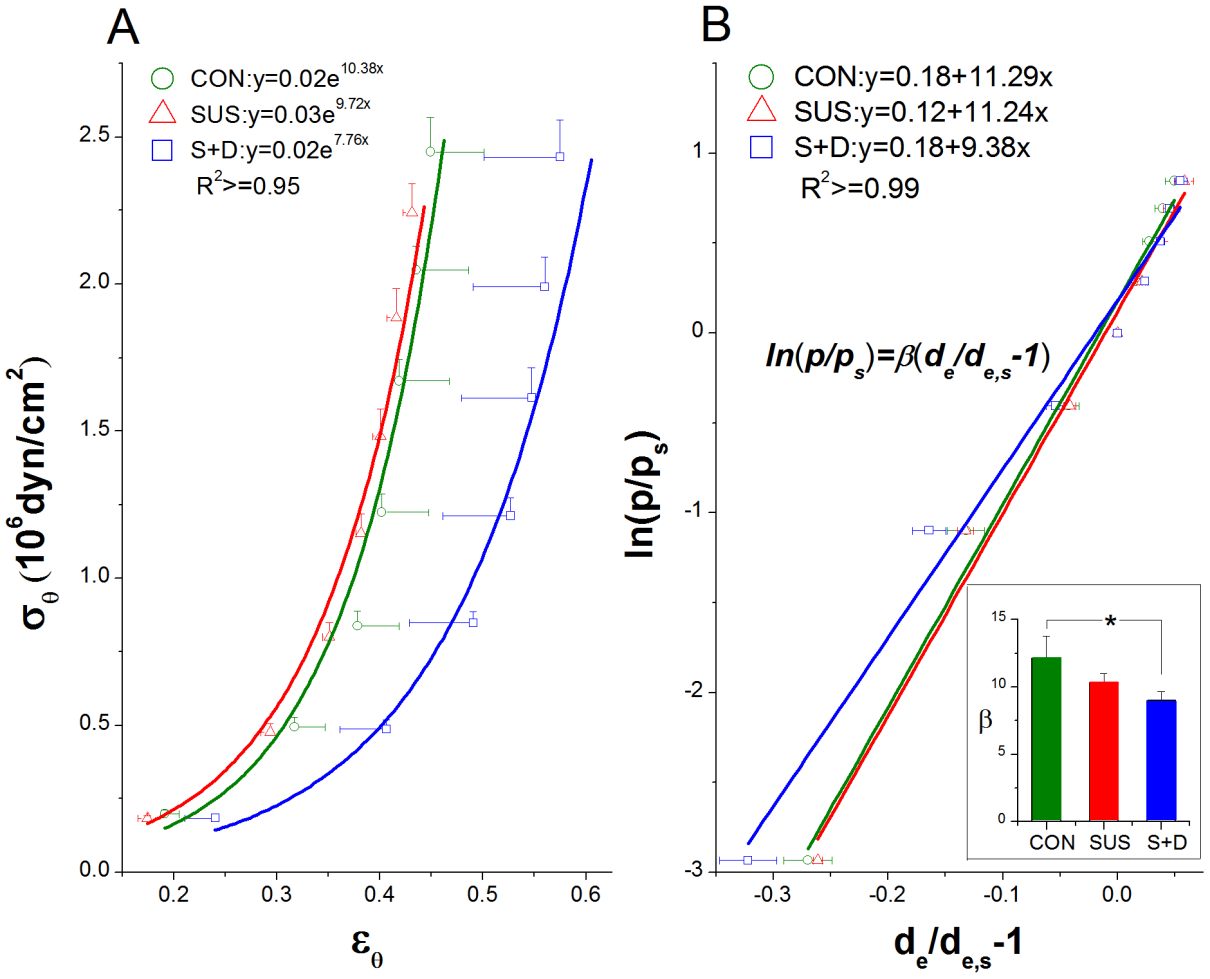
Comparison on passive mechanical behavior of middle cerebral arteries among CON, SUS, and S+D groups. (A) Circumferential stress (σ_θ_)-strain (ε_θ_) relationship for passive vessels calculated from the data of the second run. Both σ_θ_ and ε_θ_ are calculated at the external radius. Fitted curves are also shown with corresponding exponential equations and *R* ^2^ values. (B) A representative linear regression between the logarithm of pressure ratio versus the mean value of the distension ratios with the averaged values of the calculated stiffness parameter (β) being shown in the bottom right inset. The calculation was based on the data from the second run. The reference pressure (p_s_) was 75 mmHg. Values are means±SE. *n* = 8∼10 per group.*P<0.05 (1-way ANOVA).

The passive behavior exhibits typical exponential stress-strain curve with increasing stiffness at higher strains. Vessels were more distensible in S+D than that in CON and SUS vessels, as evidenced by the rightward shift of the σ_θ_–ε_θ_ curve of S+D group (Figure 3A). The decreased stiffness of the vessels is further supported by the result with overall stiffness parameter β calculated for passive vessels. The regression used to calculate β with the findings are shown in Figure 3B. The averaged β values for CON, SUS, and S+D groups were 12.1±1.7, 10.3±0.6, and 9.0±0.7, respectively, and the β value was significantly smaller in S+D compared with CON group (*P*<0.05) (Figure 3B, inset).

### Composition

**Figure 4:**
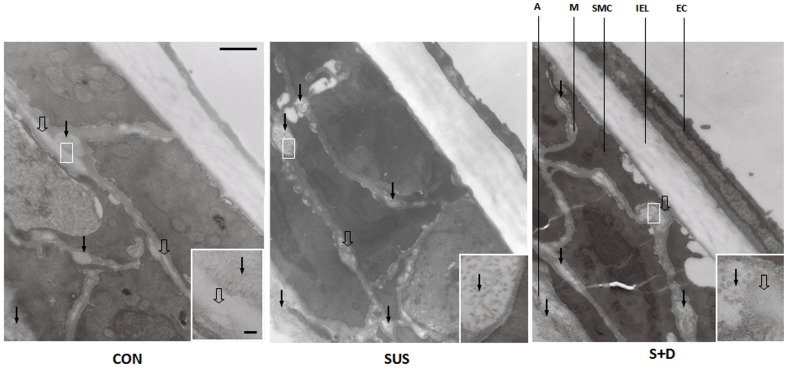
Transmission electron micrographs of the longitudinal middlemost sections of the wall of middle cerebral arteries from a CON, a SUS, and a S+D rat. Vessel specimens were prefixed in passive state at their in-vivo length under 75 mmHg for 60 min. Acellular matrix components, collagen (closed arrows) and elastin (open arrows) are clearly visible and their details is further shown in each relevant inset. Vessel histology, with regions of internal elastic lamina (IEL), media (M), and adventitia (A), and endothelial cell (EC) and smooth muscle cell (SMC) is also clearly shown. Scale bar = 500 nm. Electron micrographs in each inset, scale bar = 50 nm.

The distribution and details of collagen (closed arrows) and elastin (open arrows) are shown in Fig.4. Based on striation pattern and diameter, mainly type III collagen were observed between SMCs. Densely packed bundles of type I collagen were observed in the adventitia. Elastic fibers observed in M and A was mainly amorphous substance with varying electron-density surrounded by microfibrils at their periphery. The details of the images of collagen and elastin are further shown in the relevant insets. The morphometric data for collagen and elastin are summarized in Table 3. Most of the collagen fibrils were found in the adventitia (A) and a small amount was between SMCs in the media (M). Comparing with that of CON group, the percentage of collagen in M and A of MCAs from SUS rats increased by 68% (*P*<0.05) and 37% (*P*<0.05) and that from S+D rats increased by 64% (*P*<0.05) and 27% (*P*<0.05), respectively. And there were no significant differences in the percentage of collagen between SUS and S+D groups. Small amounts of elastin were observed in MCAs from the three groups. For SUS group, the percentage of elastin in M and A decreased significantly compared with that of CON group (*P*<0.05 or 0.01). Whereas for S+D rats, the percentage of elastin in M and A was significantly higher than that of SUS group (*P*<0.05); and in A, it is significantly less than CON group (*P*<0.05) (Table 3).

**Table 3:** Percentage of collagen and elastin in the wall of middle cerebral arteries of CON, SUS, and S+D rats.

	CON (*n* = 5)	SUS (*n* = 7)	S+D (*n* = 5)
C_M_ (%)	2.8±0.7	4.7±0.7*	4.6±0.7*
C_A_ (%)	39.3±4.4	53.9±3.2*	50.0±4.8*
E_M_ (%)	0.40±0.04	0.26±0.05*	0.42±0.15#
E_A_ (%)	1.15±0.40	0.01±0.01**	0.26±0.09* #

Values are means±SE. CON, control; SUS, 4-wk tail-suspended; S+D, 23 h/day suspension +1 h/day standing. C_M_ and C_A_ are the percentage of collagen in the media and adventitia, respectively. E_M_ and E_A_ are the percentage of elastin in the media and adventitia, respectively.

*P<0.05,

**P<0.01 vs. CON;

# *P*<0.05 vs. SUS.

## Discussion

The main findings of the present study are as follows. 1) The myogenic reactivity of MCAs is susceptible to previous pressure-induced serial constrictions. 2) During the first run of pressure steps, although the active p-d_e_ relationship of MCA is comparable between the SUS and S+D, but only the SUS, not S+D vessels, manifest strong active stiffening with lower strains. 3) In passive state, MCAs from S+D are more distensible than CON and SUS vessels. 4) SUS and S+D induce an increase and a decrease in the percentage of collagen and elastin, respectively, in the vessel wall relative to CON, whereas elastin in S+D is significantly higher than that in SUS vessels. When applied to humans, these findings support the possibility that during spaceflight microgravity may induce enhanced cerebral vasoconstriction and stronger active stiffening, which can lead to orthostatic intolerance, even though the returning astronauts do not exhibit hypotension on standing. It is also clear that circumferential stress-strain analysis, instead of pressure-diameter relationship alone, can provide a definite proof that IAG has its unique countermeasure effectiveness in preventing such an adverse effect on cerebral small resistance arteries due to microgravity exposure. There are several reasons for being sure that this is so.

**Active mechanical behavior.** In order to gain a better understanding of the myogenic mechanism and acquire the true active mechanical behavior of the isolated MCAs, three specific measures were taken in the present study. First, the experiment was designed to study the myogenic reactivity exclusively avoiding the use of any agonist as well as the KCl depolarizing solution. Second, a strict pressure increment schedule ranging between 4−175 mmHg according to [30] was adopted and repeated twice for each vessel to challenge the myogenic mechanism. Third, the circumferential stress (σ_θ_) was calculated using the Timoshenko's equation [32], since the thin-wall assumption required for the Laplace's equation is not valid for these vessels [25].

Osol et al. [30] have proposed that cerebral small artery responses to TMP may be partitioned into three phases, i.e., the initial myogenic tone (40–60 mmHg), myogenic reactivity (60–140 mmHg), and forced dilatation phase (>140 mmHg). It is noteworthy that the rightward shift of the second-run p-d_e_ curves is apparently due to the susceptibility of myogenic responsiveness to previous pressure-induced constrictions in the phase of myogenic reactivity (see Figure 1, dashed lines). This susceptibility might be an important feature of cerebral small resistance arteries, rather than simply a consequence of the previous serial pressurizations. Since even in the second run, d_e_ of both SUS and S+D was still significantly smaller than the CON group, while the pressure was increased to 175 mmHg, which is in the forced dilatation phase (see Figure 1, dashed lines). In addition, in the case for mesenteric third-order arterioles, there is no such difference in myogenic reactivity between the two runs of incremental pressure steps with the same magnitude [20]. This inherent feature may hint a unique, self-limiting protective mechanism in cerebral small resistance arteries whereby to limit the myogenic reactivity from hyper-activation and compromise the vasoconstriction whenever the SUS rat regains its normal standing posture. However, the underlying mechanisms remain to be elucidated.

Then we need to reason why during the first run of pressure increments, the active p-d_e_ relationship of SUS and S+D was very close to each other (Fig.1A, thick lines), but calculated σ_θ_ - ε_θ_ curves have shown substantial differences between these two groups (Fig. 2A, thick lines). Since σ_θ_ in Fig. 2A was calculated at the internal radius (*r*_i_ = *d*i,a/2), eq. (2) can be rewritten as: 
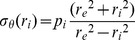
(5)


As can be seen in Table 2, *r*_i_ for SUS is different from *r*_i_ for S+D. Although the denominator seems to be the same between the SUS and S+D groups, the numerator changes considerably since both *r*_i_ and *r*_e_ for the SUS group are lower than those for the S+D. Thus, the shape of active σ_θ_–ε_θ_ curve differs substantially between these two groups. The almost parabolic σ_θ_–ε_θ_ curve within the myogenic pressure range for the SUS vessels indicates a stronger stiffening of the vessels with SMC activation resulting in smaller strains. Whereas the shape of the σ_θ_–ε_θ_ curve in the myogenic range for the S+D vessels is similar to that of CON rats, which suggests that daily 1-h –Gx intervention can effectively attenuate the active stiffening of vessels that might occur due to SUS alone. Nevertheless, at the highest pressure of 175 mmHg, the strains in MCAs from SUS and S+D still remain smaller than that of the CON rats (Fig. 2A, thick lines), which is also consistent with the axial force data (Fig. 2B). However, during the second run of pressure increments, both the p-d_e_ and the σ_θ_–ε_θ_ curves of all the three groups were shifted to the right (Figs.1A and 2A, dashed lines), showing a generalized decline in the myogenic reactivity and active stiffening after the previous serial pressure-induced constrictions. Within the myogenic range, wall stiffness of an active vessel was in the following order: SUS>CON>S+D (Fig. 2A, dashed lines). Therefore, stress-strain analysis of the active p-d_e_ data may help to reveal the unique effectiveness of IAG in attenuating the active stiffening in cerebral resistance arteries that might occur due to SUS alone. This unique feature cannot be revealed by describing solely the p-de relationship, because stress-strain analysis excludes the confounding influence of variations in vessel geometry and stress is the force per unit area and strain is related to a distension ratio. Comparing with our previous short-term study [34], it becomes clearly evident that only after a medium term simulation, daily STD can alleviate MCAs' strong active stiffness that might occur due to SUS alone. The underlying mechanisms might be related to the differential adaptation change and daily-STD countermeasure effect on cerebral versus mesenteric small resistance arteries [21], [24].

**Passive mechanical behavior.** The mechanical behavior of the passive vessels of the three groups were calculated from the second run data, since arteries in passive state may be regarded as pseudoelastic materials, exhibiting a nearly repeatable response to cyclic loading once they have been preconditioned [26]. The relative position of the passive stress-strain curves among the three groups is not consistent with that of the three p-de curves, since passive σ_θ_–ε_θ_ curves are independent of the vessel geometry. As seen in Fig. 3A, the passive σ_θ_–ε_θ_ curve for MCAs from S+D shifted rightward and showed greater variability evidenced by its greater SEs along the x-axis as compared with that from CON and SUS rats. The exponential fits to the data also indicate that the S+D vessels were the most distensible; here, the lowest exponential multiplier signifies a mild increase and thus a less steep slope. The results of stress-strain analysis also mirror the results of the β calculations. The overall stiffness parameter β, has been used as a simple and reliable parameter for describing the p-d_e_ relationship in arteries [33]. The averaged β value of rat MCAs is comparable to those reported previously [25]. As shown in Fig. 3B, the mean β value for S+D was significantly lower than that for CON (*P*<0.05). However, previous short-term simulation study shows that there are no significant differences for both the passive σ_θ_–ε_θ_ curve and the mean β value among the three groups [34]. Thus, the higher distensibility of passive vessels is also a unique adaptation change in MCAs from S+D rats gained during a medium-term simulation. The passive mechanical behavior is also essential for understanding of MCA's elasticity change due to different treatment, since elasticity implies the compliance in a uniaxial stress state. It implies that the vasodilation impairment in MCAs of SUS rats at low levels of pressure may be attenuated by the IAG countermeasure. As in the case of spontaneously hypertensive rats, the cerebral autoregulatory curve is speculated to be also shifted to the right in the SUS rats [15]–[16], [21]. When pressure is elevated, active vasoconstriction is greater in SUS; when pressure is reduced, vasodilation may be impaired in SUS than CON. However, the magnitude of this impairment may be attenuated in S+D rats, since at low levels of pressure the active stiffening of vessels is greatly attenuated (Fig. 2A) but their elasticity/compliance is significantly increased (Fig. 3A,B), which may help to preserve a greater vasodilator capacity [27].

**Acellular matrix components.** The passive σ_θ_–ε_θ_ relationship is independent of vessel geometry, thus the rightward shift of the curve for S+D vessels is presumably expected to be accompanied by a relative increase in the more elastic components of the arterial wall. Elastic fibers can easily be distended to around twice their resting length and collagen is a major non-distensible component in the arterial wall. The shape of the passive curves is generally believed to be governed at low and high pressures by elastin and collagen, respectively [26]. In this study, the percentage of collagen increased significantly in the media and adventitia of MCAs from both SUS and S+D compared with CON rats and there were no significant differences between the two groups. Whereas the percentage of elastin in the S+D was significantly higher than that of SUS group, and it tended to be less than CON group. However, the relationship of vascular acellular components and distensibility is complex and no doubt depends on factors other than proportional composition, including the distributions, orientations, and interconnections of the intramural constituents [26], [35].

**Implications.** The findings of the present study support the notion that though the enhanced myogenic reactivity and strong active stiffening of cerebral resistance arteries have an important protective function for the downstream microcirculation in microgravity [15], [18], [21], but inappropriate cerebral vasoconstriction and impaired cerebral autoregulation after long-duration space flight may compromise cerebral perfusion and lead to orthostatic intolerance upon returning to 1-G environment [9]–[11]. These findings of the present study further suggest that, in addition to the inherent, self-limiting protection mechanism, the potential effectiveness of IAG might be associated with its unique effect in alleviating active stiffening and increasing elasticity/compliance of cerebral small resistance arteries. However, further studies are needed on whether these findings can be extended and applied to humans.

Recently, a relevant paper on changes of cerebral arteries from space-flown mice has been published [36]. It reported that after a 13-day spaceflight, the myogenic responses and distensibility of mouse basilar artery were diminished and increased, respectively, with no change in medial wall thickness; and the vascular tissue stiffness of posterior communicating artery was lower relative to that of ground controls. Several factors, including species-specific differences and cabin CO_2_ level have been speculated to be responsible for the discrepancy between findings on cerebral arteries from ground SUS rats and space-flown mice [36]. To our knowledge, the greater differences in gravitationally dependent distribution of blood volume and TMP distribution along the arterial vasculature between upright humans and standing rats might be another important contributing factor [4], [37]. Nevertheless, these seem not to influence the basic conclusions on microgravity-induced adaptation in cerebral arteries thus obtained, because in the present study, the effect of redistribution of TMP on cerebral small resistance arteries that should occur in humans during microgravity is simulated by the chronic head-down tilt posture of the SUS rats.

In summary, stress-strain analysis has revealed that MCAs from medium-term (28 days) SUS rats can strongly stiffen their wall in the myogenic reactivity pressure range, which can be prevented by a countermeasure of daily 1-h –Gx gravitation (S+D). Furthermore, MCAs from the IAG countermeasure group, become more distensible than CON and SUS rats. Collagen was increased in MCAs of SUS and S+D; whereas, elastin was higher in S+D than SUS. These results have provided new insight from the biomechanical aspect to the understanding of cerebrovascular syncope-initiating mechanism for postflight orthostatic intolerance and the underlying mechanism of its prevention by IAG.

## References

[1] Sides MB, Vernikos J, Convertino VA, Stepanek J, Tripp LD, et al (2005) The Bellagio Report: Cardiovascular risks of spaceflight: implications for the future of space travel. Aviat Space Environ Med 76: 877–895. 16173686

[2] Watenpaugh DE, Hargens AR (1996) The cardiovascular system in microgravity. Handbook of Physiology: Environmental Physiology. New York: Am Physiol Soc. pp. 631–674.

[3] Zhang LF, Yu ZB, Ma J, Mao QW (2001) Peripheral effector mechanism hypothesis of postflight cardiovascular dysfunction. Aviat Space Environ Med 72: 567–575. 11396563

[4] Zhang LF (2013) Region-specific vascular remodeling and its prevention by artificial gravity in weightless environment Eur J Appl Physiol: doi:10.1007/s00421-00013-02597-00428. 10.1007/s00421-013-2597-8 23525669

[5] Hargens AR, Bhattacharya R, Schneider SM (2012) Space physiology VI: exercise, artificial gravity, and countermeasure development for prolonged space flight. Eur J Appl Physiol: 10.1007/s00421-00012-02523-00425 10.1007/s00421-012-2523-5 23079865

[6] Arbeille P, Kerbeci P, Mattar L, Shoemaker JK, Hughson R (2008) Insufficient flow reduction during LBNP in both splanchnic and lower limb areas is associated with orthostatic intolerance after bedrest. Am J Physiol Heart Circ Physiol 295: H1846–1854. 18757480 10.1152/ajpheart.509.2008

[7] Buckey JC Jr, Lane LD, Levine BD, Watenpaugh DE, Wright SJ, et al (1996) Orthostatic intolerance after spaceflight. J Appl Physiol 81: 7–18. 8828642 10.1152/jappl.1996.81.1.7

[8] Delp MD, Colleran PN, Wilkerson MK, McCurdy MR, Muller-Delp J (2000) Structural and functional remodeling of skeletal muscle microvasculature is induced by simulated microgravity. Am J Physiol Heart Circ Physiol 278: H1866–1873. 10843883 10.1152/ajpheart.2000.278.6.H1866

[9] Blaber AP, Goswami N, Bondar RL, Kassam MS (2011) Impairment of cerebral blood flow regulation in astronauts with orthostatic intolerance after flight. Stroke 42: 1844–1850. 21617145 10.1161/STROKEAHA.110.610576

[10] Gazenko OG, Genin AM, Egorov AD (1981) Summary of medical investigations in the U.S.S.R. manned space missions. Acta Astronaut 8: 907–917. 11543109 10.1016/0094-5765(81)90061-8

[11] Zuj KA, Arbeille P, Shoemaker JK, Blaber AP, Greaves DK, et al (2012) Impaired cerebrovascular autoregulation and reduced CO2 reactivity after long duration spaceflight. Am J Physiol Heart Circ Physiol 302: H2592–2598. 22492717 10.1152/ajpheart.00029.2012

[12] Young L, Yajima K, Paloski W (2009) Artificial Gravity Research To Enable Human Space Exploration. Paris: International Academy of Astronautics. pp. 1–37.

[13] Zhang LF (2001) Vascular adaptation to microgravity: what have we learned? J Appl Physiol 91: 2415–2430. 11717201 10.1152/jappl.2001.91.6.2415

[14] Hargens AR, Richardson S (2009) Cardiovascular adaptations, fluid shifts, and countermeasures related to space flight. Respir Physiol Neurobiol 169 Suppl 1S30–33. 19615471 10.1016/j.resp.2009.07.005

[15] Geary GG, Krause DN, Purdy RE, Duckles SP (1998) Simulated microgravity increases myogenic tone in rat cerebral arteries. J Appl Physiol 85: 1615–1621. 9804560 10.1152/jappl.1998.85.5.1615

[16] Osol G, Halpern W (1985) Myogenic properties of cerebral blood vessels from normotensive and hypertensive rats. Am J Physiol 249: H914–921. 4061668 10.1152/ajpheart.1985.249.5.H914

[17] Xie MJ, Zhang LF, Ma J, Cheng HW (2005) Functional alterations in cerebrovascular K(+) and Ca(2+) channels are comparable between simulated microgravity rat and SHR. Am J Physiol Heart Circ Physiol 289: H1265–1276. 15894580 10.1152/ajpheart.00074.2005

[18] Wilkerson MK, Colleran PN, Delp MD (2002) Acute and chronic head-down tail suspension diminishes cerebral perfusion in rats. Am J Physiol Heart Circ Physiol 282: H328–334. 11748078 10.1152/ajpheart.00727.2001

[19] Wilkerson MK, Lesniewski LA, Golding EM, Bryan RM Jr, Amin A, et al (2005) Simulated microgravity enhances cerebral artery vasoconstriction and vascular resistance through endothelial nitric oxide mechanism. Am J Physiol Heart Circ Physiol 288: H1652–1661. 15576439 10.1152/ajpheart.00925.2004

[20] Gao F, Cheng JH, Bai YG, Boscolo M, Huang XF, et al (2012) Mechanical properties and composition of mesenteric small arteries of simulated microgravity rats with and without daily–Gx graviation. Acta Physiol Sinica 64: 107–120. 22513459

[21] Lin LJ, Gao F, Bai YG, Bao JX, Huang XF, et al (2009) Contrasting effects of simulated microgravity with and without daily -Gx gravitation on structure and function of cerebral and mesenteric small arteries in rats. J Appl Physiol 107: 1710–1721. 19815720 10.1152/japplphysiol.00493.2009

[22] Sun B, Zhang LF, Gao F, Ma XW, Zhang ML, et al (2004) Daily short-period gravitation can prevent functional and structural changes in arteries of simulated microgravity rats. J Appl Physiol 97: 1022–1031. 15121745 10.1152/japplphysiol.00188.2004

[23] Faraci FM, Heistad DD (1990) Regulation of large cerebral arteries and cerebral microvascular pressure. Circ Res 66: 8–17. 2403863 10.1161/01.res.66.1.8

[24] Lin LJ, Bao JX, Bai YG, Zhang LF, Ma J (2009) Differential effect of simulated microgravity on myogenic tone of middle cerebral and mesenteric small arteries in rats. Acta Physiol Sinica 61: 27–34. 19224051

[25] Coulson RJ, Chesler NC, Vitullo L, Cipolla MJ (2002) Effects of ischemia and myogenic activity on active and passive mechanical properties of rat cerebral arteries. Am J Physiol Heart Circ Physiol 283: H2268–2275. 12388247 10.1152/ajpheart.00542.2002

[26] Humphrey JD (2001) Cardiovascular Solid Mechanics: Cells,Tissues and Organs. New York: Springer-Verlag.

[27] Heistad DD, Baumbach GL (1992) Cerebral vascular changes during chronic hypertension: good guys and bad guys. J Hypertens Suppl 10: S71–75. 1291659

[28] Zhang LN, Zhang LF, Ma J (2001) Simulated microgravity enhances vasoconstrictor responsiveness of rat basilar artery. J Appl Physiol 90: 2296–2305. 11356795 10.1152/jappl.2001.90.6.2296

[29] Zhang LF, Sun B, Cao XS, Liu C, Yu ZB, et al (2003) Effectiveness of intermittent -Gx gravitation in preventing deconditioning due to simulated microgravity. J Appl Physiol 95: 207–218. 12794097 10.1152/japplphysiol.00969.2002

[30] Osol G, Brekke JF, McElroy-Yaggy K, Gokina NI (2002) Myogenic tone, reactivity, and forced dilatation: a three-phase model of in vitro arterial myogenic behavior. Am J Physiol Heart Circ Physiol 283: H2260–2267. 12388265 10.1152/ajpheart.00634.2002

[31] Intengan HD, Deng LY, Li JS, Schiffrin EL (1999) Mechanics and composition of human subcutaneous resistance arteries in essential hypertension. Hypertension 33: 569–574. 9931167 10.1161/01.hyp.33.1.569

[32] Timoshenko S (1934) Theory of Elasticity. New York: McGraw-Hill.

[33] Hayashi K, Nagasawa S, Naruo Y, Okumura A, Moritake K, et al (1980) Mechanical properties of human cerebral arteries. Biorheology 17: 211–218. 7213987

[34] Cheng JH, Boscolo M, Lin LJ, Bai YG, Zhang X, et al (2009) Comparison of biomechanical behavior of cerebral and mesenteric small arteries of simulated microgravity rats. Acta Physiol Sinica 61: 386–394. 19701592

[35] Intengan HD, Schiffrin EL (2001) Vascular remodeling in hypertension: roles of apoptosis, inflammation, and fibrosis. Hypertension 38: 581–587. 11566935 10.1161/hy09t1.096249

[36] Taylor CR, Hanna M, Behnke BJ, Stabley JN, McCullough DJ, et al (2013) Spaceflight-induced alterations in cerebral artery vasoconstrictor, mechanical, and structural properties: implications for elevated cerebral perfusion and intracranial pressure. FASEB J 27: 2282–2292. 23457215 10.1096/fj.12-222687 PMC3659353

[37] Rowell LB (1993) Human Cardiovascular Control. New York: Oxford University Press.

