# Correction: Mechanics and Composition of Middle Cerebral Arteries from Simulated Microgravity Rats with and without 1-h/d –Gx Gravitation

**DOI:** 10.1371/journal.pone.0110303

**Published:** 2014-10-06

**Authors:** The PLOS ONE Staff

There is an error in Equation 1. Please see the corrected Equation 1 here.



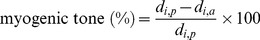
(1)

